# Supplementary material for: Predictors of time to death among cervical cancer patients at Tikur Anbesa specialized hospital from 2014 to 2019: A survival analysis
Source: PLoS One. 2022 Feb 24;17(2):e0264369. doi: 10.1371/journal.pone.0264369 (PMC8870501; doi:10.1371/journal.pone.0264369)
Supplement: Attachment — Submitted filename: Response to Reviewers.docx [file pone.0264369.s001.docx]

Date **25/12/2021**

TO- PLOS ONE

Subject: Submission of revised manuscript for publication

**Dear: Dear reviewers and editors**

We thank you for the opportunity to submit a revised version entitled “Predictors of time to death among Cervical Cancer patients at Tikur Anbesa Specialized Hospital from 2014 to 2019: A Survival Analysis “with track number PONE-D-21-03938.

**Response to Reviewers’ and Editorial comments,**

Thank you for your valuable, constructive comments and your time. We have tried to fully address all the concerns in the revised version. We are grateful for spending your valuable time on reading our manuscript. Your scientific concerns are valuable input for the improvement of this article. After carefully reading your report and the manuscript, the authors made the following revisions.

**Response to Academic editor’s comment**

1. Please ensure that your manuscript meets PLOS ONE's style requirements, including those for file naming. The PLOS ONE style templates can be found at

https://journals.plos.org/plosone/s/file?id=wjVg/PLOSOne_formatting_sample_main_body.pdf and

https://journals.plos.org/plosone/s/file?id=ba62/PLOSOne_formatting_sample_title_authors_affiliations.pdf

**Response:** We have corrected the format and the naming based on PLOS ONE's formatting sample and using the helpful links you have attached above.

2. Please review your reference list to ensure that it is complete and correct. If you have cited papers that have been retracted, please include the rationale for doing so in the manuscript text, or remove these references and replace them with relevant current references. Any changes to the reference list should be mentioned in the rebuttal letter that accompanies your revised manuscript. If you need to cite a retracted article, indicate the article’s retracted status in the References list and also include a citation and full reference for the retraction notice.

**Response:**  We have corrected the referencing. We have also added some references which are found under the updated reference section with the appropriate referencing format.

3. In the Methods, please clarify that participants provided oral consent. Please also state in the Methods:

- Why written consent could not be obtained

- Whether the Institutional Review Board (IRB) approved use of oral consent

- How oral consent was documented

- How consent was managed from patients who had died; please state whether the ethics committee waived consent in some cases.

For more information, please see our guidelines for human subjects research: <https://journals.plos.org/plosone/s/submission-guidelines#loc-human-subjects-research>

**Response:** We added all the necessary information to address the raised questions related to the reasons we have taken informed oral consent. Please see the ethical statement section line 172-183

4. In the ethics statement in the manuscript and in the online submission form, please provide additional information about the patient records/samples used in your retrospective study, including the date range (month and year) during which patients' medical records/samples were accessed.

**Response:** We added a statement **‘**patients cards were retrospectively reviewed form June,01, 2013 to June, 01, 2019 G.C to collect information regarding patients Socio-demographic, past obstetric history, medical history and received treatments.’ Please see line 180-182

5. Thank you for stating the following financial disclosure: "This study not funded by any institutions/organizations"

At this time, please address the following queries:

a) Please clarify the sources of funding (financial or material support) for your study. List the grants or organizations that supported your study, including funding received from your institution.

b) State what role the funders took in the study. If the funders had no role in your study, please state: “The funders had no role in study design, data collection and analysis, decision to publish, or preparation of the manuscript.”

c) If any authors received a salary from any of your funders, please state which authors and which funders.

d) If you did not receive any funding for this study, please state: “The authors received no specific funding for this work.”

Please include your amended statements within your cover letter; we will change the online submission form on your behalf.

**Response:** We add a financial disclosure statement: “The authors received no specific funding for this work.” (Line 329)

6. In your Data Availability statement, you have not specified where the minimal data set underlying the results described in your manuscript can be found. PLOS defines a study's minimal data set as the underlying data used to reach the conclusions drawn in the manuscript and any additional data required to replicate the reported study findings in their entirety. All PLOS journals require that the minimal data set be made fully available. For more information about our data policy, please see http://journals.plos.org/plosone/s/data-availability.

Upon re-submitting your revised manuscript, please upload your study’s minimal underlying data set as either Supporting Information files or to a stable, public repository and include the relevant URLs, DOIs, or accession numbers within your revised cover letter. For a list of acceptable repositories, please see http://journals.plos.org/plosone/s/data-availability#loc-recommended-repositories. Any potentially identifying patient information must be fully anonymized.

Important: If there are ethical or legal restrictions to sharing your data publicly, please explain these restrictions in detail. Please see our guidelines for more information on what we consider unacceptable restrictions to publicly sharing data: http://journals.plos.org/plosone/s/data-availability#loc-unacceptable-data-access-restrictions. Note that it is not acceptable for the authors to be the sole named individuals responsible for ensuring data access.

We will update your Data Availability statement to reflect the information you provide in your cover letter.

**Response:** We rephrase the data availability statement as follows ‘The collected data for this study is available at the hands of the principal author and will be available upon request. The collected data for this study are available at the hands of the principal author. The data are not shared to third party due to the reason that the data contain potentially identifying and sensitive clinical, medical and pathological information.’ (Line 327-335)

7. Your ethics statement should only appear in the Methods section of your manuscript. If your ethics statement is written in any section besides the Methods, please move it to the Methods section and delete it from any other section. Please ensure that your ethics statement is included in your manuscript, as the ethics statement entered into the online submission form will not be published alongside your manuscript.

**Response:** We accepted your recommendation and the ethics statement only appear in the methods section of our manuscript from line 171-178.

**Response to Reviewer**

1. First the wording and grammar should be improved as it is very difficult to read. I suggest a native speaker to edit this manuscript.

First the wording and grammar should be improved as it is very difficult to read. I suggest a native speaker to edit this manuscript.

**Response**: We have tried to correct all the grammatical errors using detailed revision and using online grammar checkers.

Also the introduction is not structured very well and needs to be reworked.

Response: We have made a revision on Introduction section of the manuscript.

2. Abstract
Introduction:
Authors should provide the full meaning of any abbreviations at the first use. Authors should write out the full meaning of TASH at first use. The phrase in the objective that indicate that authors assess is wrong. Authors investigated or determined. Access is not the right word to use.

**Response:** The full meaning of abbreviations at the first use is provided. The full meaning of TASH is written at first use as Tikur Anbesa specialized Hospital (TASH)

**Response:** The phrase in the objective that indicates that assess is corrected by investigated as you commented.
Methods:
The statement “..sample of 348 patients under follow-up time…” should be rephrased. Authors followed 348 women or a cohort of 348 women. Follow-up time seems vague and not standard. And it is imperative for authors use the phrase “348 patients” rather than a sample of 348 patients. This should be corrected throughout the whole manuscript.

**Response**: the phrase a sample of 348 patients is corrected by the phrase “348 patients” throughout the whole manuscript as you suggested

The sentence “The data was collected and entered using the android version CS-Entry tool and for the analysis exported to SPSS version 23” should be broken into two and made clear. Just say data was analyzed by SPSS version 23. Authors should keep their sentences simple and clear.

**Response**: The sentence is broken into two and made clear.

The data was collected using the android version CS-Entry tool. Data was analyzed by SPSS version 23.
Conclusions:
The sentences “As the stage of cancer progressed, the chance of surviving gets reduced. Therefore, due emphasis should be given on improving early detection. Routine CC screening programs for high-risk women such as elderly and HIV positive women should be strengthened” should be re written and made more clear and concise. Phrases like “surviving gets reduced” is not scientific and should be written in a better way.

**Response**: Conclusion is rewritten based on the findings in better way. The death rate of CC patients was high. The significant predictors associated with shorten time to death of CC patients were older age, advanced cancer stage at diagnosis, HIV infection and not receiving cancer treatment. Therefore, improving early detection and initiation of treatment for all CC patients is necessary in order to improve patient’s survival status. The government needs to strengthen the routine CC screening programs to address high-risk women such as elderly and HIV positive women in Ethiopia.

**Introduction**
Line 65- authors should put “early” in front of screening
The first paragraph of the introduction is succinctly written and the epidemiology is clearly stated. Therefore, I suggest authors delete their second paragraph as it only elaborately gives the epidemiology of Cervical cancer and adds no new information. It makes the introduction unnecessary long and adds no value besides the manuscript is not focused on the epidemiology of CC.
Lines 81-85 already stated in the first paragraph and must not be repeated.
Line 89: what is grandmother women? I think all grandmothers are women so no need to add women.
Line 95: WHO/ICO must be written in full at first use.
Line 103: use Ethiopia rather than “our country”
The sentence “Therefore, this study tried to assess the current survival status and predictors of time to death among CC patients in Ethiopia” is confusing. Authors investigated the current survival status and predictors of time to death among CC patients in Ethiopia and did not try to access.
I think authors should structure their introduction to make it interesting and reflect on what they seek to find. At present it is just too difficult to read and understand what they seek to do. This is my suggestion
- Define cervical cancer, its cause, its risk factors and consequences
- Brief epidemiology of cervical cancer worldwide and an emphasis on sub-Saharan Africa and in Ethiopia.
- Trends of cervical cancer deaths in Ethiopia, survival status in Ethiopia.
- What can be done to prevent deaths and what is the situation in Ethiopia
- Review literature on the predictors of time death among women with CC
- Rational for the study and the objectives

**Response:** We agreed with your suggestion to reorganize the introduction section and we have made the necessary adjustments. Through corrections made on Introduction section, we have addressed all of your comments and suggestion. We have marked newly added statements for your kind consideration from line 53-103.

**Methods**

Line 166: remove “which is found”.

**Response**: “which is found” is removed as suggested

Line 121 remove “in” in front of from

**Response**: “in” is removed as suggested.

What is the annual population of patients who received care for CC at TASH? If authors are aware of this then their sample size must be reflective of this figure.

**Response**: We couldn’t get the actual number of the annual population of patients who received care for CC at TASH. But we have tried to show the number of new cases in 2015 ‘In Ethiopia, about 7,095 new CC cases are diagnosed in 2015.’ In the third paragraph of introduction.

Why did authors adopt median survival times in calculating their sample size from a study which reported on clinical trials? This was a cohort study and so authors should explain.

**Response**: In calculating the sample size median survival times were taken from previous cohort study conducted in black lion not from clinical trials. The error was made during citing the reference.

What is “Data were collected by two BSc nurses”? please use standard statements. Data were collected by two registered nurses and not BSc nurses. In fact you can even refer to those who collected data as trained research assistants. What is the motivation to use BSc nurses and MSc nurses? Does it communicate anything?

**Response**: we corrected the statement as suggested; Data were collected by two registered nurses. BSc nurses are registered nurses. Data collection process was supervised by senior oncology nurse who has master degree on oncology nursing and who have more experience in supervising research data collection and previously participated in research data collection and supervision.

Regarding the data collection, it is imperative that authors explain succinctly how they were collected. How were the various Socio-demographic Characteristics and Past Obstetrics and medical history collected and their categorization?

**Response**: the above mentioned information was collected by reviewing the patient card. This information were written in every cancer patient cards based on the standard patient chart prepared by Ethiopian Federal Ministry of Health.

Results
Line 163: Should read “continuous variables were reported with mean and standard deviations”.

**Response**: the sentence is corrected as suggested, continuous variables were reported with mean and standard deviations

Line 181: refuse to participate not

**Response:** were refuse to participate is corrected with refuse to participate as suggested

Line 190 replace whose with who

**Response:** whose is replaced with ‘who’ as suggested
Discussion
Line 241: authors did not try but they determined or investigated.

**Response:** try to assess is corrected with investigated as suggested

First paragraph should briefly summarize the important results of the study. This must be rewritten. Must be checked for grammatical errors by a native speaker

**Response**: The important results of the study are briefly summarized in the first paragraph as requested.

Statements lie “probability of surviving gets slimed” and many other makes it difficult to read the text.

**Response:** We have tried to correct all the grammatical errors using detailed revision and using online grammar checkers.
